# Medicinal Chemistry Strategies in Targeting TGF-βR1 Kinase Domain: Unveiling Insights into Inhibitor Structure–Activity Relationship (SAR)

**DOI:** 10.3390/ph18050716

**Published:** 2025-05-13

**Authors:** Nusaiba A. Babiker, Soam Nadeem, Hasan Abu Kariem, Afra Abdul Hameed, Ahmed T. Negmeldin, Eman M. El-labbad

**Affiliations:** 1 Department of Pharmaceutical Sciences, College of Pharmacy, Gulf Medical University, Ajman 4184, United Arab Emirates; 2 Department of Pharmaceutical Organic Chemistry, Faculty of Pharmacy, Cairo University, Cairo 11562, Egypt; 3 Pharmaceutical Chemistry Department, Faculty of Pharmacy, Ain Shams University, Abbassia, Cairo 11566, Egypt

**Keywords:** TGF-β signaling pathway, TGF-βR1 kinase inhibitors, ALK5, small molecule inhibitors, kinase selectivity, structure–activity relationship (SAR)

## Abstract

The transforming growth factor-β (TGF-β) signaling pathway is involved in various cellular functions, including immunological response, extracellular matrix formation, differentiation, growth and development, and cell cycle regulation. The TGF β receptor type 1 (TGF-βR1) has emerged as a key component of this pathway, exhibiting significant overexpression in diverse malignancies, including hepatocellular carcinoma, gastric cancer, breast cancer, and colon cancer. Multiple therapeutic targets have been identified for the TGF-β signaling pathway, encompassing antibodies, ligand traps, vaccines, antisense oligonucleotides, and small-molecule TGF-βR1 kinase inhibitors. This review delineates the structural and functional characteristics of the small-molecule TGF-βR1 kinase inhibitors. The inhibitors discussed herein are categorized based on shared pharmacophoric features, notably a five-membered heterocyclic ring linked to three distinct features (R1, R2, and R3). These features interact with amino acids within the selectivity pocket, hinge region, or solvent-exposed area, respectively. These insights contribute to a clearer understanding of the structural requirements for selective TGF-βR1 inhibition. The presented findings in this review article offer a valuable foundation for future drug discovery efforts targeting the TGF-β signaling pathway.

## 1. Introduction

Transforming growth factor beta (TGF-β) is a member of an evolutionarily conserved superfamily of secreted dimeric peptide growth factors, which includes more than 30 ligands in mammals [1]. These include the TGF-β isoforms (TGF-β1, TGF-β2, and TGF-β3), as well as activins, inhibins, bone morphogenetic proteins (BMPs), growth and differentiation factors (GDFs), nodal, and Lefty [2,3]. Functionally, this superfamily governs a wide spectrum of physiological and pathological processes, including fibrosis, apoptosis, skeletal and vascular diseases, primary pulmonary hypertension, angioproliferative disorders, and cancer [4,5,6,7,8,9,10,11,12]. This review provides a comprehensive analysis of medicinal chemistry approaches to designing selective small molecule inhibitors targeting the TGF-β receptor type 1 (TGF-βR1) kinase domain. Through analyzing the structural features and corresponding biological activities of these inhibitors, we offer insights that could guide the rational design of more effective and selective TGF-βR1 kinase inhibitors. This comprehensive analysis not only consolidates the existing knowledge but also identifies gaps in the current research, thereby offering a valuable resource for future drug discovery efforts targeting the TGF-βR1 kinase domain.

## 2. TGF-β Receptor Isoforms

In mammals, TGF-β receptors appear in three isoforms: TGF-β receptor type 1 (TGF-βR1), TGF-β receptor type 2 (TGF-βR2), and TGF-β receptor type 3 (TGF-βR3). TGF-βR1 and TGF-βR2 are serine/threonine and tyrosine kinases [13]. However, TGF-βR3 receptors have no kinase activity [14]. Also, the size of TGF-βR1 ranges from 65 to 70 kDa, whereas TGF-βR2 is larger and varies from 85 to 110 kDa [15].

TGF-βR1 is recruited and phosphorylated by TGF-βR2 when it is coupled with a ligand, in which serine and threonine in the glycine–serine-rich (GS) domain are the residues where phosphorylation takes place [16]. Active TGF-βR1 phosphorylates either downstream SMAD2/3 or the kinases of noncanonical pathways [17]. TGF-βR2 is the receptor to which endogenous ligands bind and is responsible for activating the downstream signaling pathway [18]. TGF-βR2 can phosphorylate itself, TGF-βR1, or other receptors without ligand interaction; therefore, TGF-β signaling has numerous effects [19]. According to research, TGF-βR2 can be auto-phosphorylated on tyrosine amino acid side chains including Tyr259, Tyr336, and Tyr424 [20].

The most widely expressed TGF-β receptor is TGF-βR3, or betaglycan, an 849-amino acid proteoglycan that lacks kinase activity and is identified as promoting the binding of endogenous ligands to TGF-βR2 [14]. Furthermore, TGF-βR3 comprises a short cytoplasmic domain that can interact with other proteins and an extracellular domain that can cleave to a soluble extracellular domain (sTGF-βR3), which competitively inhibits the binding of TGF-β to TGF-βR2 [14].

## 3. TGF-β Signaling Pathway

**Figure 1:**
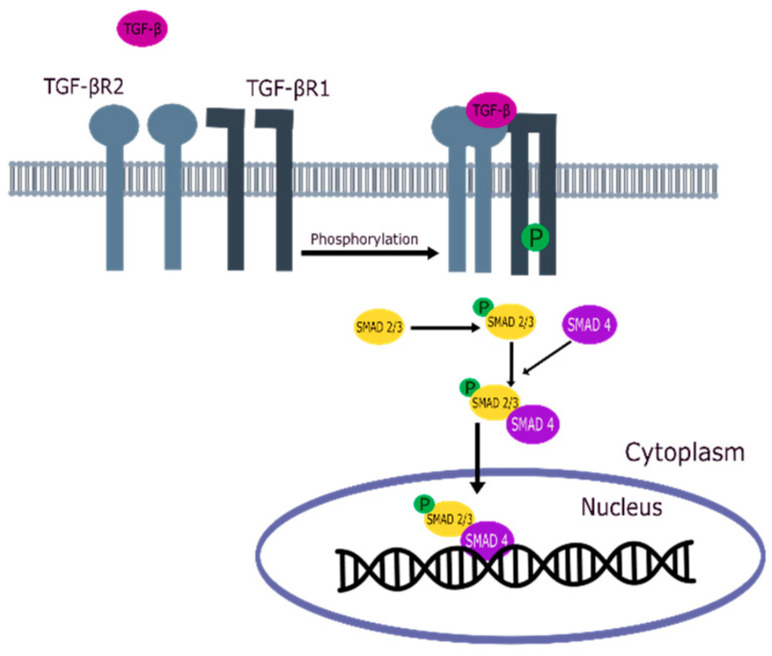
TGF-β signaling pathway.

TGF-βR1 and TGF-βR2 are key elements in activating the TGF-β signaling pathway, as shown in Figure 1 [21,22,23]. When TGF-β binds to TGF-βR2, a heterotetrameric complex is formed with TGF-βR1, which causes TGF-βR1 to be phosphorylated by TGF-βR2 [24,25]. Activated TGF-βR1 phosphorylates the SMAD2/SMAD3 complex, which shuttles into the nucleus in combination with SMAD4, recruiting many target genes for transcription with the help of other DNA-binding factors [21,26].

## 4. Role of the TGF-β Signaling Pathway in Tumor Suppression

The TGF-β signaling pathway suppresses tumor growth by several mechanisms, including indirect action through regulating cell proliferation, apoptosis, and immunological responses [27]. Malignant cell proliferation is significantly inhibited by TGF-β signaling via canonical and non-canonical pathways. By activating cyclin-dependent kinase (CDK) inhibitors p21 and p15, TGF-β blocks cell cycle progression through G1-arrest via the canonical mechanism [28,29]. Furthermore, TGF-β arrests the cell cycle in the G1 phase by suppressing nuclear factors and inhibiting DNA-binding proteins [27,29,30]. TGF-β also modulates various factors responsible for the induction of intrinsic and extrinsic apoptosis in several cell types [31]. In addition, TGF-β suppresses tumorigenesis through non-canonical pathways by triggering caspase-8-dependent programmed cell death through the p38α MAPK (mitogen-activated protein kinase) pathway and controlling the activity of immune cells, supporting cell death [27,32].

## 5. TGF-β Signaling Pathway in Tumor Promotion

In the initial phases of carcinogenesis, the TGF-β signaling pathway suppresses tumor growth, while inactivity or alterations in this pathway may occur due to mutations or the deletion of its components, resulting in tumor promotion, as observed in significant fractions of colon, pancreatic, and gastric malignancies [33,34,35,36,37,38]. Several tumor forms, including gliomas, breast, and prostate cancer, likely develop resistance to the tumor-suppressor effect of the TGF-β signaling pathway and instead utilize it to activate pathways that promote epithelial–mesenchymal transition (EMT), tumor invasion, metastatic spread, and immune system evasion [37,39,40]. EMT allows epithelial cells to acquire fibroblast-like characteristics by losing their cell polarity and specialized cell–cell interactions, facilitating their penetration into adjacent tissues, which leads to tumor invasion and metastasis [41]. Moreover, TGF-β regulates EMT, invasion, and metastatic spread while promoting tumor growth by evading the immune system [42]. TGF-β suppresses the activity of natural killer (NK) cells and exerts its immunosuppressive effects on cytotoxic T lymphocytes (CTLs) by regulating the synthesis of several pro-apoptotic factors [43].

## 6. Therapeutic Targets of the TGF-β Signaling Pathway

**Figure 2:**
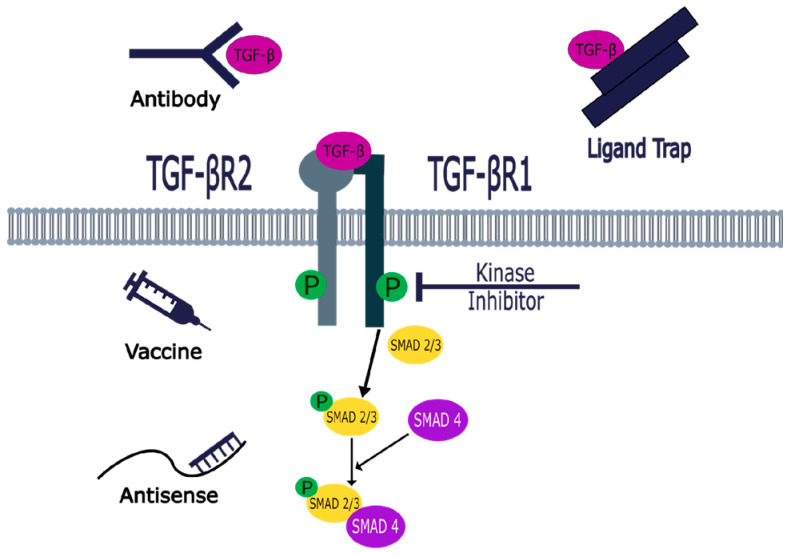
Potential therapeutic targets of the TGF-β signaling pathway.

Huang CY et al. [44] studied the approaches that can potentially target the TGF-β signaling pathway, which include ligand traps, monoclonal neutralizing antibodies, antisense oligonucleotides (ASO), vaccine-based strategy, and small molecule inhibitors, as summarized in Figure 2 [44]. The extracellular approaches, ligand traps, and neutralizing antibodies inhibit the binding of the TGF-β ligand to TGF-βR2. However, monoclonal antibodies exhibit complex structures; their tissue penetration is low, and their intertumoral uptake is limited by several barriers [44,45,46]. Regarding ligand levels, antisense oligonucleotides (ASO) are synthetic short single-stranded RNAs that correspond to specific sections of TGF-β mRNA, resulting in the breakdown of TGF-β mRNA and preventing TGF-β synthesis. However, the use of ASOs has several drawbacks: their binding affinity to RNA is difficult to predict, they may cause some adverse effects, their sequence design poses challenges, and their relatively large size can hinder plasma membrane permeability, thus affecting their transport into target cells [44]. The most common approach involves small-molecule inhibitors (SMI), which are economical, easy to produce, stable, and convenient for oral administration [44,47]. SMIs are ATP mimetics that competitively block the active site of the TGF-βR1 kinase, inhibit the phosphorylation of SMAD2/3, and subsequently inhibit the signaling pathways. Numerous small-molecule TGF-βR1 kinase inhibitors have been reported in clinical trials or pre-clinical development phases [44,45].

## 7. Exploration of TGF-βR1 Binding Site

**Figure 3:**
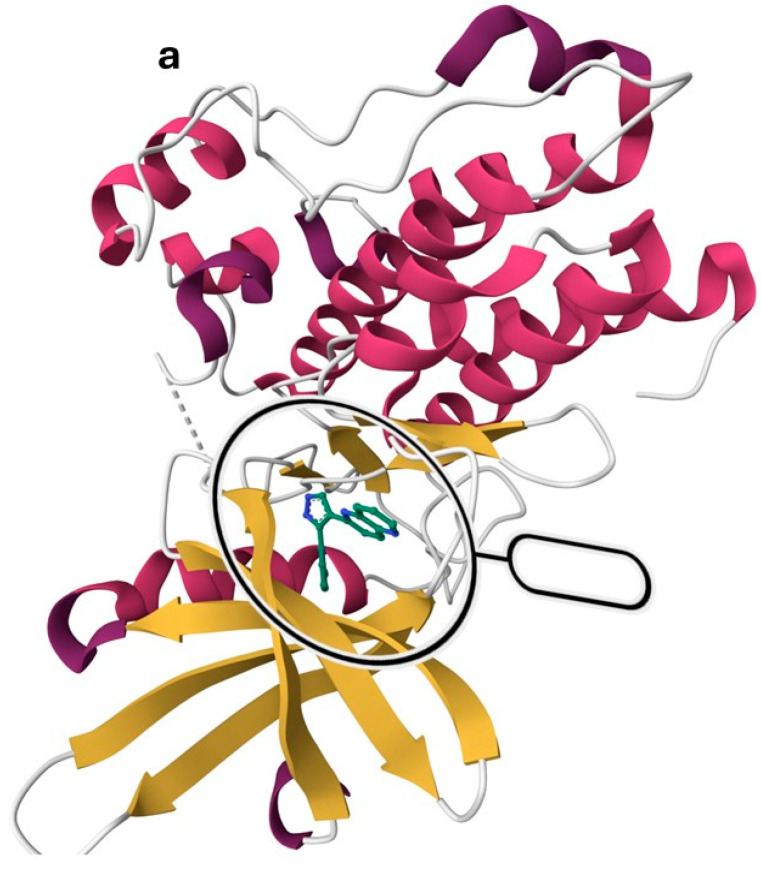

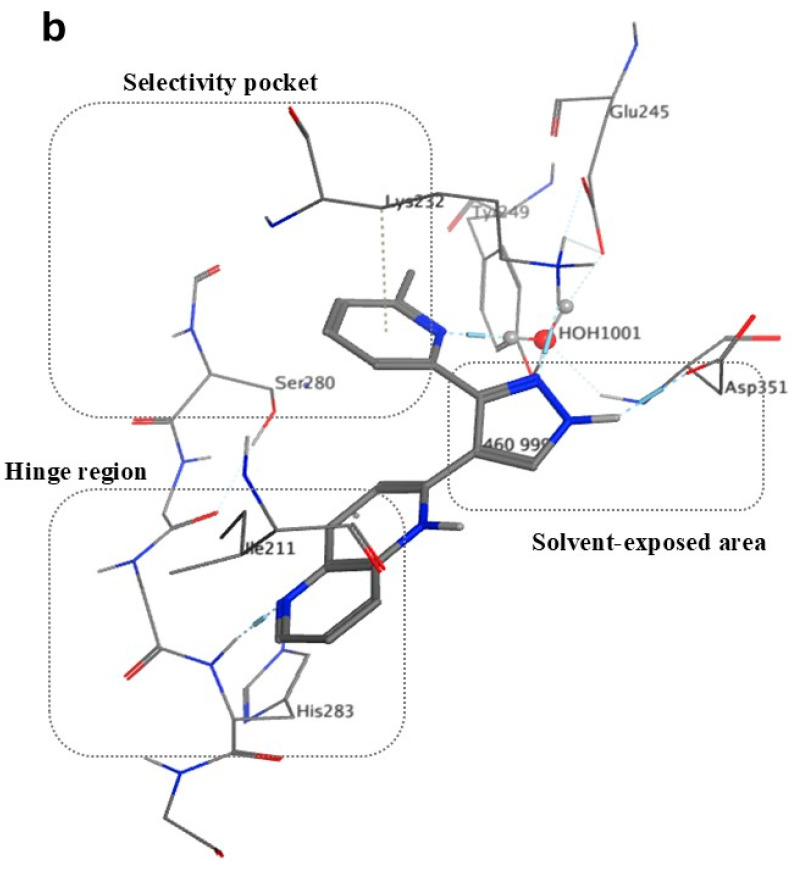
TGF-βR1 kinase domain co-crystalized with 1,5-naphthyridine conformer derivative (compound **1**) downloaded from the Protein Data Bank (PDB) ID: 1VJY [48]. (**a**) The secondary structure of TGF-βR1 was displayed with a solid ribbon. The color code indicates magenta/deep pink for helices, golden yellow for beta sheets, and grey for turns, and coils. (**b**) The binding pattern of compound 1 (shown in gray) in the TGF-βR1 kinase domain shows the selectivity pocket, hinge region, and solvent-exposed area [48].

TGF-βR1 is a one-chain protein with a sequence length of 303 amino acid residues, as shown in Figure 3a, representing the secondary structure of TGF-βR1. It was downloaded from the protein data bank (PDB) ID: 1VJY and visualized using MOE software 2020.09 [48,49]. It was co-crystalized with 1,5-naphthyridine derivative (compound **1**), which inhibited the autophosphorylation of TGF-βR1 with an IC_50_ of 6 nM. It comprises an extracellular N-terminal domain that is rich in cysteine and an intracellular C-terminal domain. The N-terminal is a single transmembrane helix homologous between TGF-βR1 and TGF-βR2 and is the pocket occupied by the TGF-β ligand [50]. TGF-βR1 structurally differs from TGF-βR2 as it contains a regulatory segment called the GS region in the C-terminal, which is essential for receptor activation [50,51].

The hinge region comprises the backbone of amino acids (residues 281–283), which connect the N-terminal to the C-terminal. Normally, the adenine moiety of ATP fits in the hinge region [48]. As shown in Figure 3b, the hinge region is occupied by the 1,5-naphthyridine scaffold of Compound **1**. Ser-280 forms a pocket in the protein’s interior called the “selectivity pocket” that is orthogonal to the hinge region [48,52]. ATP does not occupy this pocket. However, as illustrated in Figure 3b, this pocket is occupied by the pyridine moiety of compound **1**. Therefore, Ser-280 is probably the crucial protein involved in inhibitor selectivity. Thus, the selectivity pocket’s size and accessibility are controlled by this residue [48].

**Figure 4:**
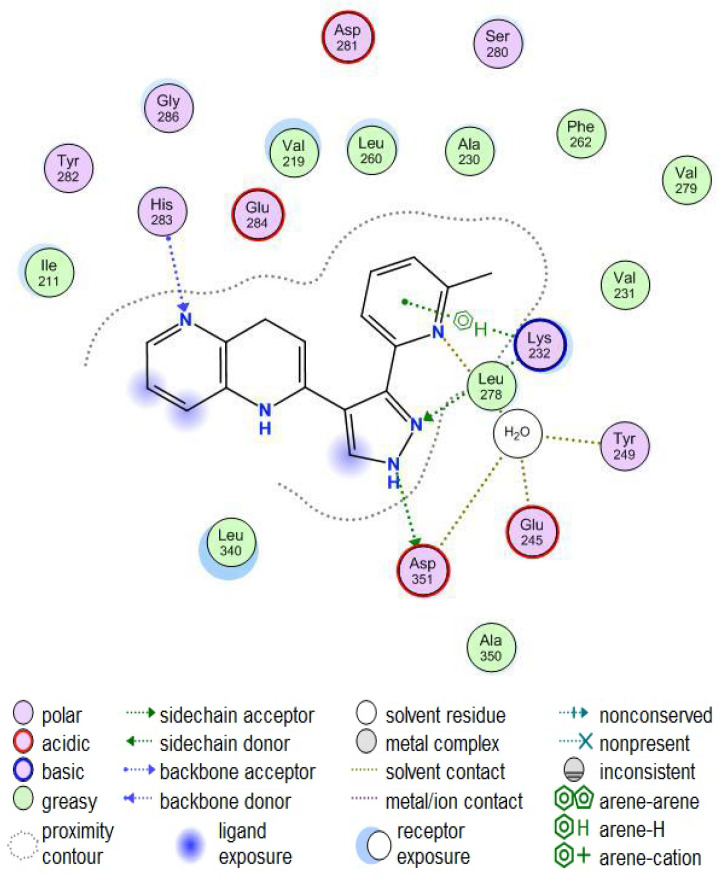
X-ray crystal structure of human TGF-βR1 kinase domain occupied by 1,5-naphthyridine derivative (compound **1**) (visualized using MOE software [49]); 2D interactions are highlighted with dotted lines.

Compound **1**, as shown in Figure 4, binds in the kinase domain of the TGF-βR1, in which N5 is linked to the backbone N of His-283. Two hydrogen bonds linked the pyrazole NH to the side chain of Asp-351 and N2 to the side chain of Lys-232. A hydrogen bond links the Pyridine N1 to a water molecule, which is also connected to Tyr-249, Glu-245, and Asp-351, explaining why the presence of a hydrogen bond acceptor in this site is crucial. It was also found that, to optimize the interaction, there must be a small electron-withdrawing group linked to the phenyl or pyridine moieties, since there are two interactions in the selectivity pocket: one with the pyridyl’s N1 and the electrostatic interaction between Ser-280 oxygen and the ring of the ligand [48].

## 8. Reported TGF-βR1 Inhibitors

**Figure 5:**
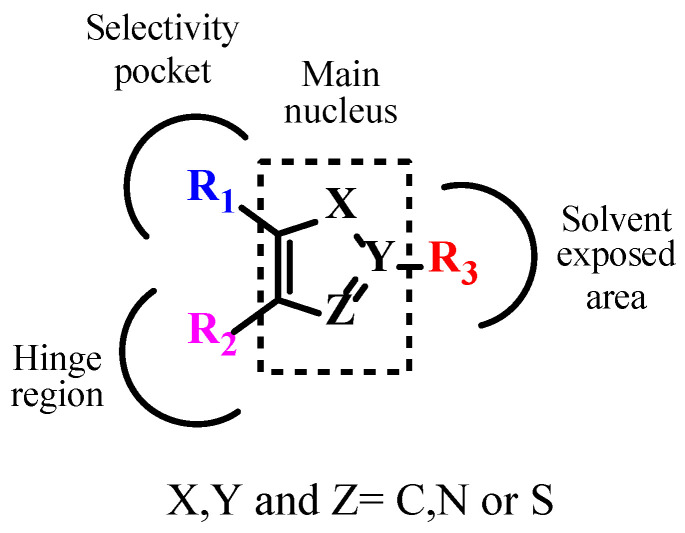
Pharmacophoric features of TGF-βR1 kinase inhibitors.

The literature searches of Google Scholar (https://scholar.google.com/, accessed on 5 July 2023) accessed on 5th July 2023, for relevant articles were performed using the key term combinations of ‘TGF-βR1 kinase inhibitors’, ‘transforming growth factor beta receptor 1 kinase inhibitors’, and ‘ALK5 kinase inhibitors. Our literature review of the reported TGF-βR1 kinase inhibitors revealed a common pharmacophoric feature, described in Figure 5. These features include a central core scaffold of a five-membered heterocyclic ring as a bio-isostere of the adenine ring of the natural substrate ATP. The five-member heterocyclic core scaffold was substituted with three features (R1), (R2), and (R3) to interact with amino acids in the selectivity pocket, hinge region, or solvent-exposed area, respectively.

In the current section, the reported inhibitors will be described based on the core scaffold nucleus, namely imidazole, pyrazole, thiazole, triazole, and miscellaneous. The rationale behind this classification is to facilitate a systematic comparison of the reported inhibitors. The evaluation will involve a comparison of the reported inhibitors’ design, binding mode, and activity against TGF-βR1.

### 8.1. Imidazole Derivatives

**Figure 6:**
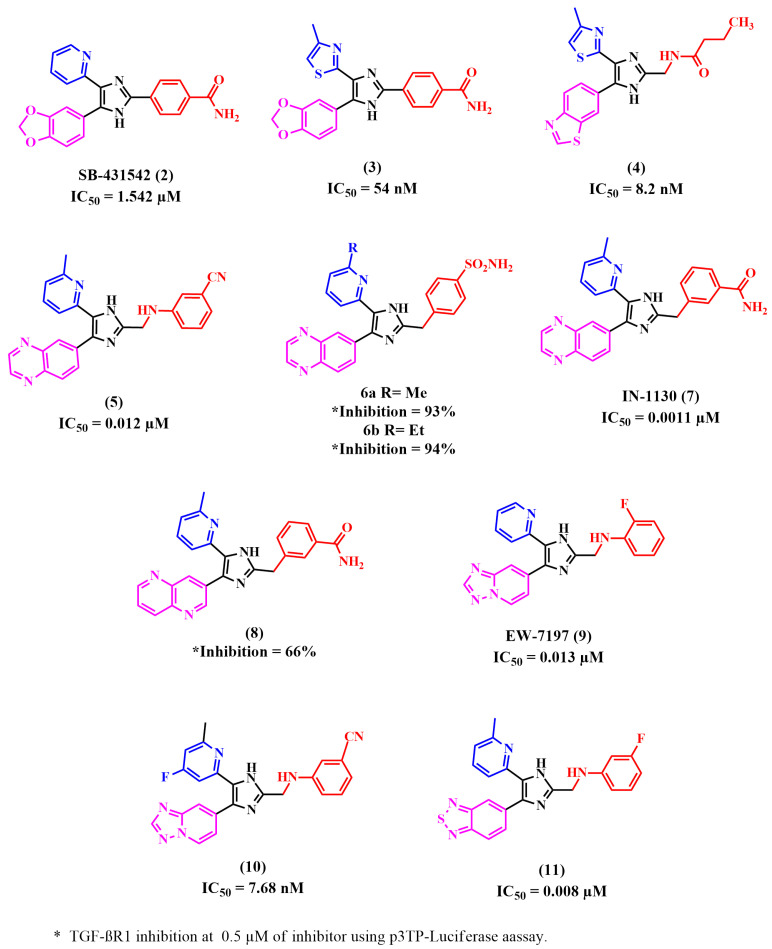
Reported TGF-βR1 kinase inhibitors with imidazole as the main nucleus.

Many reported TGF-βR1 kinase inhibitors possessed an imidazole moiety as the central nucleus scaffold, as illustrated in Figure 6.

(SB-431542) Compound **2** is a well-reported ATP-competitive TGF-βR1 inhibitor initially developed for treating progressive fibrosis [53,54,55]. The literature reported that a typical water-mediated H-bond interaction usually forms between the 2-pyridyl group of compounds **2** and Tyr-249, Glu-245, and Asp-351 amino acids [48,53,54,55,56,57,58,59,60,61]. Amada et al. [62] replaced the 2-pyridyl moiety with a thiazolyl group to provide a novel series of 4-thiazolylimidazoles, assuming that the thiazolyl group would form similar binding interactions. The results revealed that compound **3**, whose thiazole ring was substituted with a methyl group in the 4-position, showed the highest potency when compared with compounds with other substituents. Therefore, it was considered an initial lead compound, and its benzamide moiety was modified by alklyamide or aliphatic amines. The results suggested that functional groups that have no basicity, like the benzamido or butanamido group, will show better potency than aliphatic amines with strong basicity. Also, the potency is influenced by the length of the alkylamide linkage and its position. Therefore, butanamide analogs showed decreased potency since they had a longer linker. Moreover, studying the imidazole ring’s 5-position substitution demonstrated that 4-hydroxyphenyl analog had low membrane permeability, explaining why it showed high potency in enzyme inhibition and no effect on cells. Compound **4**, possessing a 1,3-benzothiazol-6-yl moiety, exhibited high potency in both assays. The most potent compound is compound **4**, which has an IC_50_ value of 8.2 nM for enzyme inhibitory activity and 32 nM for the TGF-β-induced inhibition of SMAD2/3 phosphorylation at the cellular level. To study compound 4’s binding interactions in the ATP binding site of TGF-βR1, it was docked into the molecular model, which showed a network of hydrogen bonds between the nitrogen atom of the 4-methylthiazol-2-yl with the Asp351 NH backbone, hydroxy H of Tyr249, and carboxy O of Glu-245 mediated by a water molecule [62].

In addition, Kim et al. [53] used (SB-431542) compound **2** as a lead compound to establish a series of 4(5)-(6-alkylpyridin-2-yl)imidazoles substituted at the 2-position of the imidazole ring with a phenylaminomethyl or phenylmethylamino moiety possessing a carbonitrile or carboxamide group at the imidazole’s 2-position. They also replaced the benzo[1,3]dioxolyl with the quinoxalinyl group. The compound series was then synthesized and evaluated utilizing a purified human TGF-βR1 kinase domain enzyme assay and a cell-based assay. The results showed that all the quinoxalinyl analogs were more potent than the corresponding benzo[1,3]dioxolyl analogs. A meta-position substituted with carbonitrile or a carboxamide functionality showed increased inhibitory activity compared to substitution in the para-position. Also, an increased TGF-βR1 inhibition was observed with the presence of the carbonitrile group compared to the carboxamide, probably due to their better cellular permeability. Quinoxalinyl analog **5** showed the highest potency regarding TGF-βR1 inhibitory activity, with an IC_50_ of 0.012 µM, compared to **2**, which inhibited TGF-βR1 with an IC_50_ of 1.542 µM. Flexible docking studies of compound **5** showed a good fit in the TGF-βR1 kinase domain. The quinoxaline ring maintained a primary H-bond interaction with the His283 residue of the hinge region [53].

Furthermore, Kim et al. investigated the effect of replacing the carboxamide group with sulfonamide, as well as introducing a methylene or ethylene linker between the phenyl ring central imidazole and phenyl ring. They designed a series of 4-(6-alkylpyridin-2-yl)-5-(quinoxalin-6-yl)imidazole derivatives. It was found that ethylene linkage enhanced the activity compared to methylene linkage, while it decreased the activity for the n-propyl linkage. They also found that activity was reduced by substituting the pyridyl ring at the 6-position with a group bulkier than the ethyl group. Among this series, compounds **6a** and **6b** showed 93% and 94% TGF-βR1 inhibition at 5 µM in a luciferase reporter assay [63].

In addition, pharmacokinetic studies of (IN-1130) compound **7**, a highly potent TGF-βR1 kinase inhibitor, showed that the 2- or 3-position of the 6-quinoxalinyl moiety undergoes metabolic oxidation, leading to a significant decrease in its activity [64]. Based on the above-mentioned findings, Kim et al. designed novel 4(5)-(6-methylpyridin-2-yl)imidazole and pyrazole derivatives. They attempted to examine the ability of the 6-quinolinyl or 1,5-naphthyridin-2-yl moiety to maintain the N-1 in the 6-quinoxalinyl of compound **7**, which was found to be crucial for binding interactions in the TGF-βR1 kinase domain. They also aimed to observe the position of the substituted benzoyl moiety on the central nucleus that leads to the best TG1 inhibition. The results showed that the 6-quinolinyl group had higher inhibitory activity than the 1,5-naphthyridine-2-yl moiety. Also, 6-quinolinyl imidazole derivatives had better activity than the corresponding pyrazole derivatives. Pharmacokinetic studies showed that the major metabolite of compound **8**, the most active compound, with 66% inhibition of TGF-βR1 at 0.05 µM, was much smaller when compared to the major metabolite of compound **7** [65].

Maddeboina Krishnaiah et al. found that (EW-7197) compound **9** is a selective, highly potent TGF-βR1 kinase inhibitor with an IC_50_ of 13nM [66]. Moreover, Bonafoux et al.’s research indicated that substituting the methyl-2-pyridyl moiety at the 5-position with a fluoro group improved the potency and selectivity for TGF-βR1 inhibition [59]. Therefore, Maddeboina Krishnaiah designed a series of 5-(3-,4-, or 5-fluoro-substituted-6-methylpyridin-2-yl)-4-([1,2,4]triazolo[1,5-a]pyridin-6-yl)imidazoles. They attempted to design derivatives of compound **9** possessing fluoro substituents in different positions to assess their effect on TGF-βR1 inhibition. They also evaluated the selectivity of the synthesized compounds by assessing their inhibitory activity against p38α MAP kinase, whose kinase domain shows the highest similarity to the TGF-βR1 kinase domain. The results demonstrated that 3-fluoro- and 5-fluoro-substituted pyridine ring derivatives exhibited a comparable potency level against TGF-βR1 to compound 9. Nevertheless, the substitution of the pyridine ring with a 4-fluoro group decreased the inhibitory activity of TGF-βR1 and p38α MAP kinase more than **9** and affected the chemical stability. Compound **10** was the most potent and showed higher potency than compound 9, with an IC_50_ value of 7.68 nM in a kinase assay and 82% inhibition at 100 nM in a kinase assay and luciferase reporter assay, [67].

Correspondingly, Zhen Guo et al. found that the high oral bioavailability of compound **9** is because of the [1,2,4]triazolo[1,5-α]pyridin-6-yl scaffold, since it has two adjacent nitrogen atoms, making it possible for metabolic oxidation to take place in the 2-position of this moiety [68,69]. Thus, they hypothesized that inserting a more fat-soluble group, such as benzo[c][1,2,5]thiadiazole or thieno[3,2-c]pyridine at the 4-position of the imidazole would produce comparable TGF-βR1 inhibitory activity [68,69]. Among the designed series, compound **11** had the highest activity (IC_50_ = 0.008 μM) [68].

### 8.2. Pyrazole Derivatives

**Figure 7:**
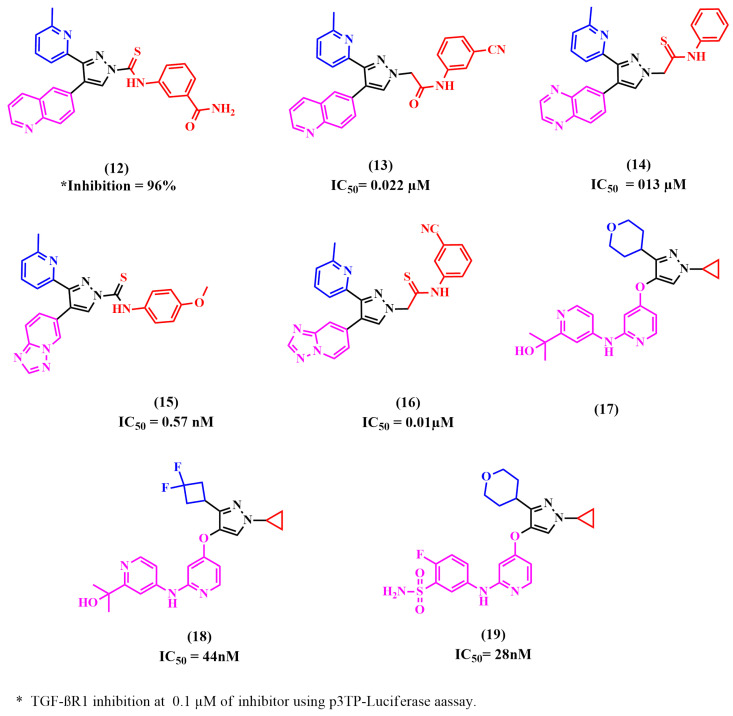
Reported TGF-βR1 kinase inhibitors possessing pyrazole as the main nucleus.

The primary nucleus scaffold of numerous reported TGF-βR1 kinase inhibitors was the pyrazole moiety, as shown in Figure 7.

Dewang et al. reported that the TGF-βR1 inhibition activity is increased by linking a carbonitrile- or carboxamide-substituted phenyl ring to a five-membered heterocyclic ring via carbothiomide, aminomethylene, or methyleneamino linkage, as in compound **7** (IN-1130) [53,63,64,65,70,71,72,73,74,75,76]. Based on these results, they designed a new series possessing a phenyl group with a carbonitrile or carboxamide substitution, connected to 2-pyridyl-substituted pyrazole and imidazole derivatives. For comparison, a 4-quinolinyl moiety was substituted for the 6-quinolinyl moiety of **7** since this was among the most appropriate warhead groups [53]. In the p3TP-luciferase reporter experiment, all the pyrazole derivatives with a carbothioamide linkage showed more than 85% inhibition at 0.1 M. As anticipated, the inhibitory activity of TGF-βR1 was enhanced through the addition of a carboxamide group at the meta-position of the phenyl ring. TGF-βR1 inhibition was significantly higher in the pyrazole derivatives with a carbothioamide linkage than in the equivalent compounds with a methylene linkage. The pyrazole derivatives with a methylene linkage exhibited greater potency in comparison to their corresponding imidazole derivatives. TGF-βR1 inhibition was greater in compounds with a 6-quinolinyl moiety on the central pyrazole ring than in those with a 4-quinolinyl moiety. Using cell-based luciferase reporter assays, the synthesized compounds were assessed for their TGF-βR1 inhibitory activity. The most effective compound was **12**, which showed 96% and 93% inhibition at 0.1 µM in luciferase reporter assays using HaCaT cells transiently transfected with a p3TP-luc reporter construct and ARE-luc reporter construct, respectively [77].

Jin et al. found that a pyrazole ring and a phenyl ring linked with a thioamide linkage were unstable during long-term storage and slowly broke down to a pyrazole ring. Consequently, they designed a series of compounds possessing amidomethylene, amidoethylene, thioamidomethylene, or thioamidoethylene linkages, which are more stable. All the synthesized compounds were then biologically evaluated using a purified human TGF-βR1 kinase domain assay. The TGF-βR1 inhibitory activity was increased in compounds possessing pyrazoles linked by an aminomethylene or thioamidomethylene linkage to meta-carbonitrile or carboxamide-substituted phenyl ring. In contrast, inhibitory activity was decreased in meta-substituted compounds with amidoethylene or a thioamidoethylene linkage compared to unsubstituted compounds, suggesting that longer linkages and a substituent hinder the binding of compounds possessing them. The selectivity profile of all prepared compounds was assessed using p38α MAP kinase. The results revealed that compound **13** inhibited TGF-βR1 activity (IC_50_ = 0.022 µM) and showed 84% inhibition at 0.1 µM in a luciferase reporter assay. It also showed the highest selectivity, with an index of >45 [78].

In addition, Jin et al. attempted to enhance the stability of the linkage between the central pyrazole and the phenyl ring through using the thioamidomethylene linkage instead of the thioamide linkage. They also examined the effect of a longer alkyl chain in the linkage on the inhibition activity. The results showed that the amidoethylene linkage decreased the potency by 2.2-fold compared to the amidomethylene linkage. Previous studies by Jin et al. showed that phenyl or benzyl moieties, linked to the central heterocyclic ring possessing meta-carbonitrile or carboxamide, significantly increased TGF-βR1’s inhibitory activity. However, the results revealed that the unsubstituted pyrazole derivatives of this series had higher potency than the corresponding carboxamide-substituted and carbonitrile-substituted derivatives. Additionally, the selectivity profile of the compounds for TGF-βR1 vs. p38α MAP kinase showed that the synthesized compounds had high selectivity; compound **14** was the most selective and most potent (IC_50_ = 013 µM) [79].

As a continuation of the efforts to design a more potent TGF-βR1 inhibitor, Jin et al. reported a novel series of 1-substituted-3-(6-methylpyridin-2-yl)-4-([1,2,4]triazolo[1,5-a]pyridin-6-yl)pyrazoles as potential TGF-βR1 inhibitors by replacing the quinoxalin-6-yl or quinolin-6-yl moieties with a [1,2,4]triazolo[1,5-a]pyridin-6-yl moiety and introducing various substituents in the phenyl ring. The findings demonstrated a considerable increase in TGF-βR1 inhibitory activity and selectivity with the insertion of the [1,2,4]triazolo [1,5-a]pyridin-6-yl moiety and the phenycarbo-thioamido moiety at the 4- and 1-positions of the pyrazole ring, respectively. Compared to the unsubstituted compound, the 4-OMe substituted compound showed five-fold more potency, whereas the 4-CN substitution did not improve in terms of potency. TGF-βR1 inhibition in meta-substituted compounds was slightly higher for 3-Cl- and 3-Ome-substituted compounds. On the other hand, ortho-substituted compounds showed no effect. Among the new series, compound **15**, the most potent, inhibited TGF-βR1 phosphorylation with an IC_50_ value of 0.57 nM and showed 94% inhibition at 100 nM in a luciferase reporter assay [80].

Metabolic oxidation is less experienced with [1,2,4]triazolo[1,5-a]pyridin-6-yl moiety since it has two neighboring nitrogen atoms [81]. Therefore, Jin et al. used this moiety to design imidazole and pyrazole derivatives to replace the quinoxalin-6-yl scaffold. An attempt was also made to evaluate the effect of methyleneamido or methylenethioamido linkage and various substituents in the phenyl ring on the TGF-βR1 inhibitory activity. Compounds possessing a methylenethioamido linkage showed better potency. The inhibitory activity of the compounds possessing a methylenethioamido linkage was enhanced through substituting the phenyl ring with 2-F, 3-CN or 3-CONH_2_. It was concluded that compound **16** showed the highest inhibitory activity on the TGF-βR1 phosphorylation, with an IC_50_ value of 0.01 µM [81].

Guofeng Xu et al. used (LY-3200882) **17**, a selective TGF-βR1 inhibitor, as a lead compound and designed 4-(pyridin-4-oxy)-3-(3,3-difluorocyclobutyl)-pyrazoles to explore the binding site and try to access extra binding pockets through the introduction of different side chains. Initially, they studied the modification of substituents of the tetrahydro-2H-pyran scaffold in **17,** since it readily underwent oxidative metabolism. The results showed that the introduction of a phenyl group slightly improved inhibitory activity, although electron-donating or electron-withdrawing groups performed similarly. However, the inhibitory action of derivatives with amide and carboxyl groups was decreased, probably due to their high polarity or hydrogen-donating properties. Most N-heterocycle substituent derivatives were typically well tolerated. Furthermore, aliphatic substituents displayed a similar pattern when evaluated. Compound **18**, which contains 1,1-difluorocyclobutyl, was 2-fold more potent than **17**. Therefore, 1,1-difluorocyclobutyl was fixed in further changes. Then, several substitutions of the tertiary alcohol of compound **17** were evaluated. The results suggested that potency is reversely affected by hydrophobicity. In the pharmacokinetics profiles, the most effective compounds showed increased plasma exposure compared to **17**, which may have been caused by 1,1-difluorocyclobutyl’s greater metabolic stability. Overall, compound **18** was the most potent compound, with an IC_50_ of 44 nM and 65.7% tumor growth inhibition in a CT26 xenograft mouse model [82].

Another study by Tan et al. studied the binding mode of compound **17**, revealing that a strong H-bond is displayed by N-cyclopropyl-1H-pyrazole, forming a water bridge, which was important for binding efficacy. Thus, hydroxyl-substituted pyridine was replaced by aryl, aromatic, and aliphatic heterocyclic derivatives to obtain novel 4-((1-cyclopropyl-3-(tetrahydro-2H-pyran-4-yl)-1H-pyrazol-4-yl)oxy)pyridine-2-yl)amino derivatives. The results revealed that carboxylic substitution significantly decreased potency in the kinase assay. Moreover, no activity was seen when piperidine was substituted for the pyrazole and pyridine rings. The phenlysulfonamide moiety enhanced both the oral bioavailability and the inhibitory efficacy. Compound **19** demonstrated high TGF-βR1 inhibition (IC_50_ = 28 nM), and the pharmacokinetics research revealed an oral bioavailability of 57.6% [83].

### 8.3. Thiazole Derivatives

**Figure 8:**
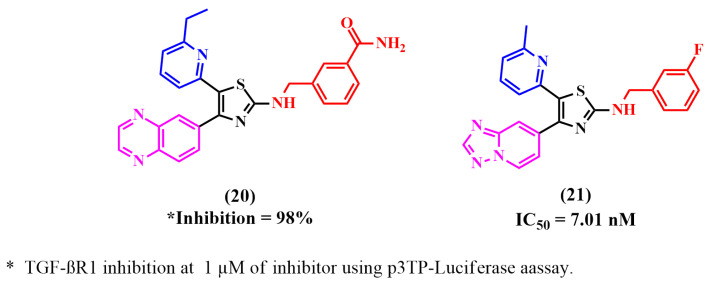
Reported TGF-βR1 kinase inhibitors with thiazole as the main nucleus.

As illustrated in Figure 8, numerous reported TGF-βR1 kinase inhibitors had thiazole as the main nucleus scaffold.

Kim et al. established new 5-(pyridin-2-yl)thiazole analogs with thiazole as the central nucleus scaffold and attempted to evaluate the effect of a meta- or para-carbonitrile- or carboxamide substitution on the phenylmethyl-amino group at thiazole ring’s 2-position on the TGF-βR1 inhibition. 6-methylpyridyl derivatives showed significantly high TGF-βR1 inhibition rates, but the 6-ethylpyridyl derivatives showed similar or lower activity. To enhance the TGF-βR1 inhibition, the meta-position for the carbonitrile and carboxamide substitution was optimal. The most potent compound, **20**, showed high inhibition for TGF-βR1 at 1µM (p3TP-luciferase, 98%) [71].

Maddeboina Krishnaiah et al. developed a series of 2-benzylamino-4(5)-(6-methylpyridin-2-yl)-5(4)-([1,2,4]triazolo[1,5-a]pyridin-6-yl)thiazoles by linking the thiazole and phenyl ring with an aminomethylene linkage. They attempted to investigate whether the amino group forms an extra binding interaction with the ATP binding site. In these compounds, they replaced the quinoxalin-6-yl or quinoline-6-yl group at the 5-position of thiazole with a [1,2,4]triazolo[1,5-a]pyridin-6-yl moiety to overcome the observed metabolic oxidation, as [1,2,4]triazolo[1,5-a]pyridin-6-yl scaffold is thought to be less susceptible to oxidation because of the two adjacent nitrogen atoms. SAR studies revealed that the best TGF-βR1 inhibition was obtained by introducing a substituent in the phenyl ring, such as the 3-F or 4-F group, allowing the disubstituted derivatives to be extended. Compound **21**, the most potent compound, inhibited TGF-βR1 phosphorylation (IC_50_ = 7.01 nM) and 61% inhibition at 30 nM in a luciferase reporter assay [69].

### 8.4. Triazole Derivatives

**Figure 9:**
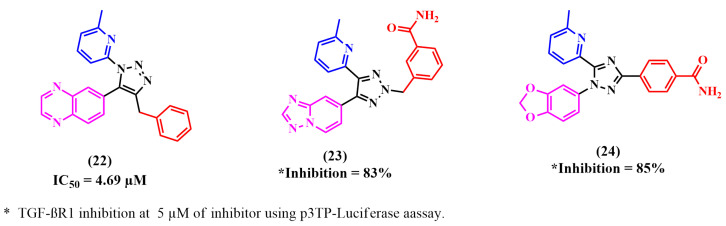
Reported TGF-βR1 kinase inhibitors with triazole as the main nucleus.

Many reported TGF-βR1 kinase inhibitors possessed a pyrazole moiety as the main nucleus scaffold, as illustrated in Figure 9.

Fei Li et al. relied on the fact that a 1,2,3-triazole scaffold can replace flat heteroaromatic rings. Moreover, this can be readily produced using ‘click chemistry’ and can easily be attached to various substituents [84,85]. Thus, they designed 1-(6-methylpyridin-2-yl)-5-(quinoxalin-6-yl)-1,2,3-triazole derivatives as potential TGF-βR1 inhibitors by substituting the 1,2,3-triazole ring at the 4-position to study its ability to form an active conformation. TGF-βR1 inhibition was enhanced by alkyl-substituted 1,2,3-triazole. However, relatively low TGF-βR1-inhibitory activity was shown by aryl-substituted 1,2,3-triazoles due to their unfavorable fit with the ATP binding pocket of TGF-βR1. Compound **22** showed the highest potency, with an IC_50_ of 4.69 µM [70].

Later, Kim et al. designed two novel series of (2-pyridyl)[1,2,3]-triazole derivatives by adding a methyl or ethyl moiety to the triazole ring at the 2-position. Among the synthesized compounds, compound **23** showed significant TGF-βR1 inhibition (SBE-luciferase activity, 25%; p3TP-luciferase activity, 17%) at a concentration of 5 µM [70].

As mentioned, the kinase domain of p38α MAP kinase has the highest similarity with the TGF-βR1 kinase domain. A selective inhibitor of p38α MAP kinase is 4-pyridyl-substituted triaryl-imidazoles [52,72,86]. In addition, a recent study by Callahan et al. reported that 2-pyridyl-substituted triaryl imidazoles, including (SB-431542) **2**, are significant inhibitors of TGF-βR1 compared to p38α MAP kinase [55]. Using these findings, Kim et al. designed a series of 2-pyridinyl[1,2,4]triazoles and assessed the TGF-βR1 inhibition of these compounds. Although all the compounds showed high selectivity for TGF-βR1 and almost no inhibitory activity for p38α MAP kinase, only compound **24** exhibited comparable inhibitory activity to compound **2**. The selectivity of compound **24** for TGF-βR1 versus p38α MAP kinase depends on the 2-pyridinyl moiety, since its nitrogen forms a H-bond with the OH of Ser280. Then, the designed compounds were synthesized, and luciferase reporter assays were used to evaluate their TGF-βR1 inhibitory activity. The carboxamide derivative **24** showed significant TGF-βR1 inhibition and much lower inhibition against p38α MAP kinase (p3TP-Luciferase, 85% at 5 µM and 4% at 10 µM, respectively) [72].

### 8.5. Miscellaneous

**Figure 10:**
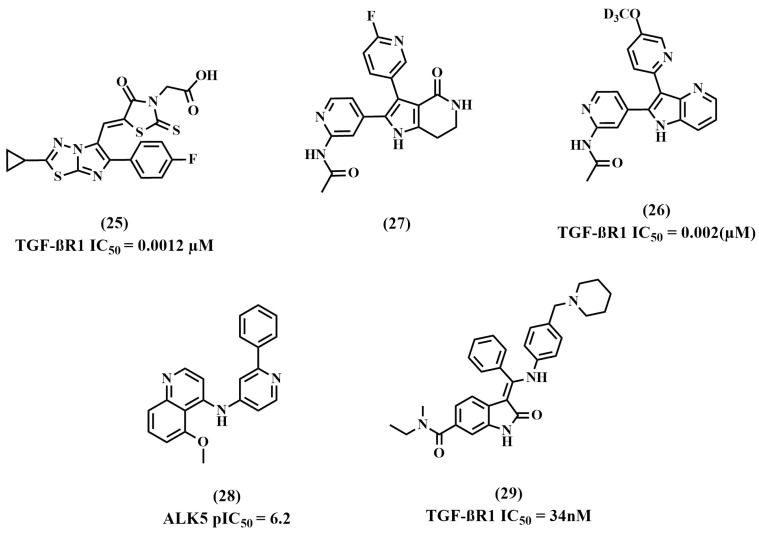
Miscellaneous TGF-βR1 kinase inhibitors.

Patel et al. reported a new series of TGF-βR1 inhibitors utilizing the reported imidazo[2,1-b][1,3,4]thiadiazoles scaffold. SAR studies revealed that substituting the C-5 of imidazo[2,1-b][1,3,4]thiadiazoles with rhodamine acetic acid was optimal for its activity in cyclopropyl-substituted compounds. Further analysis showed that one electro-withdrawing group at the C-6 position maintains this TGF-βR1 inhibition, while adding several groups reduces the inhibitory activity. The designed compounds were evaluated using a TGF-β-induced SMAD2/3 phosphorylation cell-based assay. Compound **25**, Figure 10, the most potent compound, showed prominent TGF-βR1 inhibition (IC_50_ = 0.0012 µM) [87].

Yong Zhang et al. studied the binding interactions of compound **26** to the TGF-βR1 kinase domain in a high-resolution crystal structure. Several interactions were confirmed, including the hydrogen bonds between the pyridinylacetamide group and His283, the N-fluoropyridine and the Tyr249, Glu245, and Asp351 residues, and the lactam amide and Lys232 and Asp351 residues [88,89,90]. Yong Zhang et al. aimed to design 4-azaindole analogs by allowing several ring substitutions and replacements in the pyrrololactam of compound **26** while maintaining the above interactions [90]. To investigate the hydrophobic region of the kinase pocket, they prepared a library of 3-pyridyl azaindoles, and as predicted, some of the non-polar substitutions, such as the replacement of fluorine with a methoxy group on the pyridine scaffold, maintained the inhibitory activity for TGF-βR1. Also, better selectivity was obtained through inserting bulkier substituents in the pyridine ring. The designed compounds were biologically evaluated using a murine tumor model comprising anti-PD1 antibodies. Compound **27** showed significantly higher anti-tumor efficacy when combined with an anti-mouse-PD-1 antibody than monotherapy in a murine tumor model. Additionally, compound **27** potently inhibited SMAD protein expression in NHLF and primary human T cell assays [90].

Sabat et al. screened the internal chemical collection and defined compound **28** as a lead compound with respectable potency in the TGF-βR1 enzymatic assay (TGF-βR1 pIC_50_ = 6.2). Compound **28** was docked into the TGF-βR1 protein’s kinase domain. The binding interactions included an H-bond between the quinoline ring’s nitrogen and the His283 residue of the kinase’s hinge region. In this pose, a possible pseudo-H-bond could form between the carbonyl oxygen of Asp281 and the quinoline ring’s C-H at position C2. More hydrogen bonds were formed between compound **28**’s pyridyl and Lys232’s side chain and the conserved water molecule that Asp351, Glu245, and Tyr249 coordinated. The phenyl group, connected to the pyridine ring in its C2 position, is then pointed toward the back hydrophobic pocket. Sabat et al. designed new 4-substituted quinolines using the hypothesized binding insights, which have better inhibitory activity. They also attempted to evaluate the role of the hydrophobic pocket groups in TGF-βR1 inhibition. The designed compounds showed good TGF-βR1 enzyme and cellular potency but a high clearance rate. Therefore, the series was substituted with a correlated 7-substituted-pyrazolo[4,3-b]pyridine to reduce the lipophilicity of the phenyl ring. Although these compounds showed significant TGF-βR1 kinase inhibition, this was coupled with cardiac toxicity, leading to project termination [91].

Roth et al. performed a high-throughput screening in an internal drug collection to identify TGF-βR1 inhibitors using TGF-βR1 substrate phosphorylation assays. Compound **29** and a few other indolinone derivatives displayed two-digit nM potency in the TGF-βR1 assay. Compound **29**’selectivity was identified in a kinase selectivity panel since it was reported alongside other indolinones chemotypes in earlier kinase projects and demonstrated a promising selectivity profile. Therefore, it was chosen as a lead compound [92,93,94]. The binding interactions of indolinones in the recognized kinase ATP domain were confirmed via X-ray crystallography, which showed that the amido moiety’s binding to the specificity pocket, formed by gatekeepers Phe262 and Lys232, might be responsible for **29**’s excellent selectivity profile. However, the fundamental side chain in compound **29** is directed to the water phase, indicating that there is space for structural changes in this area. SAR confirmed that the highest TGF-βR1 inhibition and selectivity profile was exhibited in 6-amido-substituted derivatives compared to unsubstituted or 5-amido-substituted compounds. In general, 4-aryl-substituted compounds displayed good potency, while neutral compounds showed less potency. Also, the results revealed that the insertion of ethylamido or ethylmethylamido moiety at the 6-position of the indolinones showed higher potency and better activity. These compounds were synthesized and biologically assessed for their ability to inhibit the TGF-βR1-mediated signal transduction in a cellular setting. Many compounds showed IC_50_ for one-digit nM and no inhibition at 1 μM for other kinases, and are therefore considered promising TGF-βR1 inhibitors [94].

## 9. Conclusions

**Figure 11:**
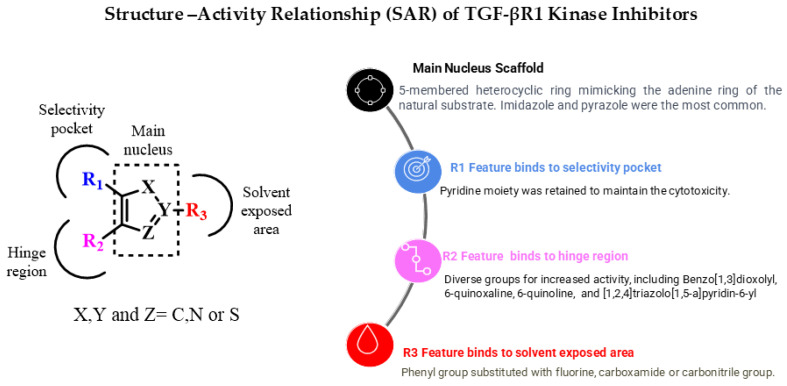
Overview of TGF-βR1 kinase inhibitors’ structure–activity relationship.

Currently, inhibiting the TGF-β signaling pathway is of great interest to researchers due to its potential role in cancer treatment. In this context, designing small-molecule inhibitors that target the kinase domain of TGF-βR1 is a widely used strategy to develop anti-cancer agents, since SMIs have numerous advantages. In this work, a combination of a computational drug design approach and rational medicinal chemistry concepts was used to evaluate reported TGF-βR1 kinase inhibitors in a systematic manner, combining knowledge obtained through a 3D analysis of the binding site of TGF-βR1 kinase and the crystal structures reported in Figure 3 and Figure 4. In addition, the structural characteristics and associated biological activities of reported compounds **1**–**29** were analyzed based on the pharmacophoric features described in Figure 5. This systematic approach led to the conceptualization of the structure–activity relationship illustrated in Figure 11.

The reported TGF-βR1 kinase inhibitors possessed a five-membered heterocyclic ring as a bio-isostere of the adenine ring of the natural substrate, representing the main core scaffold. Imidazole and pyrazole were the most commonly reported scaffolds. Three features—(R1), (R2), and (R3)—were added to the five-membered heterocyclic scaffold that interacted with amino acids in the selectivity pocket, hinge region, or solvent-exposed region, respectively. We also discussed the effect of various substituents and functional groups on the cytotoxic activity of the reported TGF-βR1 kinase inhibitors. Most of the reported compounds retained the pyridine moiety as an R1 feature connected to the main scaffold to maintain the cytotoxic activity, as in compounds **2**,**5**–**16** and **20**–**24**. However, numerous small functional groups on this pyridine group were evaluated for their effect on the inhibitory activity. While R2, in most of the reported compounds that showed relatively increased TGF-βR1 inhibitory activity, ranged between 6-quinoxaline (compounds **5**,**6a**,**6b**,**7**,**14**,**20**,**22**), which is the most commonly found moiety, [1,2,4]triazolo[1,5-a]pyridin-6-yl (compounds **9**,**15**,**16**,**21**,**23**), benzo[1,3]dioxolyl (compounds **2**,**3**,**24**), and 6-quinoline (compounds **12**,**13**). Furthermore, the R3 feature that showed high potency for TGF-βR1 kinase inhibition was mainly a phenyl group with various substituents, including carboxamide (compounds **2**,**3**,**7**,**8**,**12**,**20**,**23**,**24**), carbonitrile (compounds **16**,**13**), or fluorine (compounds **21**,**9**). Compound **15**, which showed the highest TGF-βR1 inhibitory activity among the reported TGF-βR1 kinase inhibitors studied in this article, with an IC_50_ value of 0.57 nM, possessed a pyrazole as the main core scaffold, connected to a pyridine, [1,2,4]triazolo[1,5-a]pyridin-6-yl and 3-OMe-phenyl moiety. In conclusion, this review aims to provide a consolidated framework that may assist researchers in identifying future opportunities for drug discovery targeting the TGF-βR1 kinase domain.

## Abbreviations

The following abbreviations are used in this manuscript:

ALK5	Activin receptor-like kinase 5
ASO	Antisense oligonucleotide
ATP	Adenosine triphosphate
CDK	Cyclin-dependent kinase
CN	Carbonitrile
C-H	Carbon–hydrogen
CTLs	Cytotoxic T lymphocytes
DNA	Deoxyribonucleic acid
EMT	Epithelial–mesenchymal transition
EW-7197	TGF-βR1 kinase inhibitor (compound 9)
GS	Glycine-serine-rich
H-bond	Hydrogen bond
IC_50_	Half maximal inhibitory concentration
IN-1130	TGF-βR1 kinase inhibitor (compound 7)
MAPK	Mitogen-activated protein kinase
MOE	Molecular operating environment
NHLF	Normal human lung fibroblast
NK	Natural killer (cells)
PD-1	Programmed cell death protein 1
PDB	Protein Data Bank
R1, R2, R3	Variable substituent groups in a molecule
RNA	Ribonucleic acid
SAR	Structure–activity relationship
SBE	SMAD binding element
SMAD	Homolog of mothers against decapentaplegic (a family of signaling proteins)
SMI	Small molecule inhibitor
sTGF-βR3	Soluble transforming growth factor beta receptor type 3
TGF-β	Transforming growth factor beta
TGF-βR1	Transforming growth factor beta receptor type 1
TGF-βR2	Transforming growth factor beta receptor type 2
TGF-βR3	Transforming growth factor beta receptor type 3
